# Temporal perceptual training enhances visual acuity in adult amblyopia: a single-case study

**DOI:** 10.1038/s41433-026-04688-7

**Published:** 2026-06-29

**Authors:** Aaron Khodami, Luca Battaglini

**Affiliations:** Department of General Psychology, University of Padua, Padua, Italy

**Keywords:** Medical research, Scientific community

## Introduction

Amblyopia is usually framed as a persistent spatial-acuity disorder, yet temporal processing is also abnormal, including impaired visual synchrony and prolonged temporal integration [1, 2]. This raises a mechanistic question: if residual acuity loss is partly constrained by temporal-resolution limits, temporal perceptual learning may improve acuity. Although perceptual learning can modify adult visual performance [3], most amblyopia protocols train spatial features. We therefore tested whether two-flash fusion training, a temporal discrimination task, transfers to monocular visual acuity.

## Methods

**Fig. 1:**
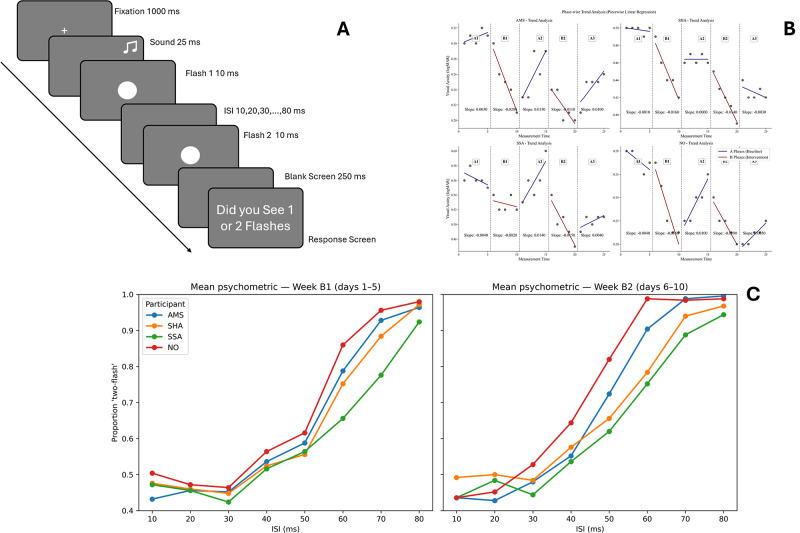
Task Design and Results. **A** Two-flash fusion task used during intervention phases. Each trial began with fixation, followed by an auditory cue, two brief flashes separated by a variable inter-stimulus interval, and a single/two-flash response. **B** Phase-wise piecewise linear trends. Negative slopes during intervention phases indicate progressive acuity improvement during temporal training. **C** Mean two-flash psychometric functions during the first and second intervention blocks. Curves show the proportion of ‘two-flash’ responses across inter-stimulus intervals from 10 to 80 ms.

Four adults with amblyopia completed a replicated ABAB single-case experimental design [4] followed by delayed follow-up. Each phase contained five daily monocular logMAR acuity measurements with the fellow eye patched. Baseline phases contained no temporal training. Intervention phases contained one 400-trial two-flash fusion block per day. Each trial presented two 10-ms Gaussian flashes separated by inter-stimulus intervals of 10–80 ms with 10 ms steps. The design conceded 25 acuity observations and 4000 temporal-training trials per participant (See Fig. 1A). Outcomes were summarised by phase means, Tau-U, non-overlap indices, piecewise trend estimates and two-flash psychometric performance.

## Results

Visual acuity improved during intervention phases in all participants and was retained at follow-up (Fig. 1B; Table 1). Mean logMAR decreased from initial baseline to the second intervention by 0.067, 0.078, 0.052 and 0.052 for Participants. Final adjusted Tau-U values ranged from −0.53 to −0.86, indicating lower acuity thresholds during intervention phases after trend correction. Piecewise slopes were generally negative during intervention phases and positive or smaller during baseline phases. Temporal performance also improved: B1-to-B2 psychometric functions shifted upward, mean two-flash accuracy increased and fitted 50% fusion thresholds decreased by 4.1–15.6 ms (See Fig. 1C).

**Table 1:** Participant-level clinical essentials, phase outcomes, single-case statistics and temporal-training indices.

Measure	AMS	SHA	SSA	NO
**Clinical essentials**				
Age/sex	25/M	32/F	26/F	26/F
Amblyopia subtype	Strabismic	Strabismic +anisometropic	Anisometropic	Strabismic
Amblyopic eye	R	L	R	R
Baseline Snellen acuity	20/40	20/63	20/80	20/32
**Visual-acuity phase means, logMAR**				
A1, initial baseline	0.308	0.498	0.562	0.272
B1, first intervention	0.252	0.450	0.528	0.232
A2, reversal baseline	0.260	0.460	0.552	0.237
B2, second intervention	0.241	0.420	0.510	0.220
FU, delayed follow-up	0.240	0.426	0.524	0.224
Δ A1–B2	−0.067	−0.078	−0.052	−0.052
Δ A1–FU	−0.068	−0.072	−0.038	−0.048
**Tau-U non-overlap and trend-adjusted effects**				
A vs B	−0.96*	−0.98*	−1.00*	−0.88*
Trend A	0.45	−0.32	−0.60	−0.67
Trend B	−1.00*	−0.95*	−0.26	−0.95*
A vs B − Trend A	−0.71*	−0.61*	−0.55	−0.40
A vs B + Trend B	−0.87*	−0.92**	−0.74*	−0.80**
Final adjusted Tau-U	−0.86**	−0.76**	−0.53*	−0.57*
**Piecewise visual-acuity trend model**				
Overall trend term	0.003	−0.001	−0.004	−0.004
Slope during B1	−0.023**	−0.015***	0.002	−0.012***
Slope during A2	0.012	0.001	0.018**	0.014***
Slope during B2	−0.014*	−0.013**	−0.011	−0.005
Slope during FU	0.007	−0.002	0.008	0.009**
Model *R*²	0.915	0.972	0.902	0.962
Adjusted *R*²	0.864	0.955	0.843	0.939
**Two-flash fusion training**				
Daily accuracy, first to last session	0.603–0.700	0.615–0.703	0.585–0.655	0.655–0.745
Δ daily accuracy	+0.097	+0.088	+0.070	+0.090
Mean accuracy, B1	0.643	0.634	0.599	0.677
Mean accuracy, B2	0.689	0.675	0.638	0.730
Δ mean accuracy, B2–B1	+0.045	+0.041	+0.039	+0.053
T₅₀, B1 threshold, ms	39.6	40.8	41.7	34.4
T₅₀, B2 threshold, ms	35.5	25.1	36.5	29.0
Δ T₅₀, B2–B1, ms	−4.1	−15.6	−5.3	−5.4
Overall two-flash mean	0.666	0.655	0.618	0.704

A1 = initial baseline; B1 = first intervention; A2 = reversal baseline; B2 = second intervention; FU = follow-up. Negative logMAR changes indicate improved acuity. Negative Tau-U values indicate lower logMAR values during intervention phases. Negative T₅₀ changes indicate reduced fitted 50% two-flash fusion threshold from B1 to B2. *p < 0.05 for Tau-U analyses; **p < 0.001 for Tau-U analyses. For piecewise regression slopes, *p < 0.05, **p < 0.01 and ***p < 0.001. Significance markers are descriptive within this single-case framework and should not be interpreted as population-level efficacy evidence.

The table is transposed to emphasise within-participant patterns. Clinical detail is limited to information necessary for interpreting heterogeneity in amblyopia type and baseline severity.

## Discussion and conclusion

This Brief Communication provides single-case evidence that temporal perceptual learning can coincide with improvement in adult amblyopic visual acuity. The findings do not establish population-level efficacy and require masked controlled replication. Their value is mechanistic: across heterogeneous amblyopic cases, a temporal task produced phase-linked acuity gains, supporting the hypothesis that temporal-resolution mechanisms contribute to residual spatial vision loss in adult amblyopia [5].

## Data Availability

Participant-level visual-acuity and two-flash fusion data will be made available upon reasonable request to the corresponding author.

## Code availability

Analysis code will be made available upon reasonable request to the corresponding author.
